# Supplementary material for: Empirical methods for controlling false positives and estimating confidence in ChIP-Seq peaks
Source: BMC Bioinformatics. 2008 Dec 5;9:523. doi: 10.1186/1471-2105-9-523 (PMC2628906; doi:10.1186/1471-2105-9-523)
Supplement: Additional File 1 — A variety of html documents from the USeq web site detailing the available applications, their best usage, output file type descriptions, command line menus, etc. [file 1471-2105-9-523-S1.zip › USeqUserGuides/JavaDoc.zip!/JavaDoc/util/bio/parsers/gff/GadFlyGffExtractor.html]

GadFlyGffExtractor


|  |  |  |
| --- | --- | --- |
| **Overview** \| **Package** \| **Class** \| **Use** \| **Tree** \| **Deprecated** \| **Index** \| **Help** | |  |
| **PREV CLASS**   **NEXT CLASS** | **FRAMES**    **NO FRAMES**     **All Classes** |  |
| SUMMARY: NESTED \| FIELD \| CONSTR \| METHOD | DETAIL: FIELD \| CONSTR \| METHOD |  |


---


## util.bio.parsers.gff Class GadFlyGffExtractor

```
java.lang.Object
  util.bio.parsers.gff.GadFlyGffExtractor
```

---

``` public class GadFlyGffExtractor extends java.lang.Object ```

Class for building annotation objects from GffFeatures, basically a converter, specific to the old dmel GadFly annotation.
GffFeature object are built from the following type of gff file.
Assumes a specific order of incoming features:
(exon(s), translation(optional), transcript) many then a gene/transposon/rRNA etc
example gff gadfly format
2R gadfly exon 67118 67499 . + . genegrp=CG8416; transgrp=CG8416-RD; name=CG8416:8
2R gadfly exon 68758 68907 . + . genegrp=CG8416; transgrp=CG8416-RD; name=CG8416:3
2R gadfly exon 69442 69522 . + . genegrp=CG8416; transgrp=CG8416-RD; name=CG8416:4
2R gadfly exon 69585 70954 . + . genegrp=CG8416; transgrp=CG8416-RD; name=CG8416:6
2R gadfly translation 67344 69773 . + . genegrp=CG8416; transgrp=CG8416-RD
2R gadfly transcript 67118 70954 . + . genegrp=CG8416; transgrp=CG8416-RD; name=CG8416-RD
2R gadfly gene 66411 70954 . + . genegrp=CG8416; name=CG8416; dbxref=GO:0016318; dbxref=GO:0007391; dbxref=GO:0007391; dbxref=GO:0007391; dbxref=GO:0007010; dbxref=GO:0003931; dbxref=GO:0016318; dbxref=GO:0007369; dbxref=GO:0007254; dbxref=GO:0007164; dbxref=GO:0003931; dbxref=GO:0003924; dbxref=GO:0003931; dbxref=GO:0007405; dbxref=GO:0030239; dbxref=GO:0016203; symbol=Rho1; dbxref=FlyBase:FBgn0014020; cytorange=52E3-52E4; cdna\_clone=GH20776; cdna\_clone=LD03419
2L gadfly exon 47514 52519 . + . genegrp=TE19092; transgrp=TE19092-RA; name=TE19092:1
2L gadfly transcript 47514 52519 . + . genegrp=TE19092; transgrp=TE19092-RA; name=TE19092-RA
2L gadfly transposable\_element 47514 52519 . + . genegrp=TE19092; name=TE19092; symbol=jockey{}277; dbxref=FlyBase:FBti0019092; cytorange=21A3-21A3
2L gadfly exon 1791017 1792026 . - . genegrp=CR31930; transgrp=CR31930-RA; name=CR31930:1
2L gadfly exon 1790806 1790958 . - . genegrp=CR31930; transgrp=CR31930-RA; name=CR31930:2
2L gadfly transcript 1790806 1792026 . - . genegrp=CR31930; transgrp=CR31930-RA; name=CR31930-RA
2L gadfly pseudogene 1790806 1792026 . - . genegrp=CR31930; name=CR31930; symbol=Gr22d; dbxref=FlyBase:FBgn0045498; cytorange=22B2-22B2
Assumes that there is at least one exon and transcript per gene/transposon/rRNA etc.
Assumes that start is always less than end/stop. Use orientation to get 1 or -1, (+ or -)

---

**Constructor Summary**

`GadFlyGffExtractor(java.io.File gffFile,
int startNt,
int stopNt)`


| **Method Summary** | |
| --- | --- |
| `void` | `buildExon(GffFeature f)` |
| `void` | `buildGeneGrp(GffFeature f)` |
| `void` | `buildGenericFeature(GffFeature f)` |
| `void` | `buildTranscript(GffFeature f)` |
| `void` | `buildTransGrp()` |
| `void` | `buildTranslation(GffFeature f)` |
| `java.util.ArrayList` | `getGeneGrps()` |
| `java.util.LinkedHashSet` | `getGenericFeatureHash()`             Contains all the feature types that were not recognized by the GadFlyGFFExtractor. |
| `java.util.ArrayList` | `getGenericFeatures()` |
| `boolean` | `isGenericFeaturesFound()` |
| `java.lang.String` | `toString()` |

**Methods inherited from class java.lang.Object**

`equals, getClass, hashCode, notify, notifyAll, wait, wait, wait`

**Constructor Detail**

### GadFlyGffExtractor

```
public GadFlyGffExtractor(java.io.File gffFile,
                          int startNt,
                          int stopNt)
```


**Method Detail**

### toString

```
public java.lang.String toString()
```

:   **Overrides:**: `toString` in class `java.lang.Object`

---


### getGenericFeatureHash

```
public java.util.LinkedHashSet getGenericFeatureHash()
```

:   Contains all the feature types that were not recognized by the GadFlyGFFExtractor.
    Should be user added items like CRMs, enhancers, etc.

---


### getGenericFeatures

```
public java.util.ArrayList getGenericFeatures()
```

---


### buildGenericFeature

```
public void buildGenericFeature(GffFeature f)
```

---


### buildExon

```
public void buildExon(GffFeature f)
```

---


### buildTranslation

```
public void buildTranslation(GffFeature f)
```

---


### buildTranscript

```
public void buildTranscript(GffFeature f)
```

---


### buildTransGrp

```
public void buildTransGrp()
```

---


### buildGeneGrp

```
public void buildGeneGrp(GffFeature f)
```

---


### getGeneGrps

```
public java.util.ArrayList getGeneGrps()
```

---


### isGenericFeaturesFound

```
public boolean isGenericFeaturesFound()
```


---


|  |  |  |
| --- | --- | --- |
| **Overview** \| **Package** \| **Class** \| **Use** \| **Tree** \| **Deprecated** \| **Index** \| **Help** | |  |
| **PREV CLASS**   **NEXT CLASS** | **FRAMES**    **NO FRAMES**     **All Classes** |  |
| SUMMARY: NESTED \| FIELD \| CONSTR \| METHOD | DETAIL: FIELD \| CONSTR \| METHOD |  |


---
